# Acute peripheral ischemia in healthy female patient: an indirect and unanticipated diagnosis of spontaneous thrombus in the aortic arch

**DOI:** 10.1186/s13019-020-01337-2

**Published:** 2020-10-01

**Authors:** Laura Rings, Igor Schwegler, Nestoras Papadopoulos, Achim Häussler, Dragan Odavic, Magdalena Schmidt, Omer Dzemali

**Affiliations:** 1 Department of Cardiac Surgery, Triemli Hospital Zurich, Birmensdorferstrasse 497, CH-8063 Zürich, Switzerland; 2 Department of Vascular Surgery, Triemli Hospital Zurich, Zürich, Switzerland; 3 Institute for Radiology and Nuclear Medicine, Triemli Hospital Zurich, Zürich, Switzerland

**Keywords:** Aortic arch, Thrombus, Peripheral embolization, Selective unilateral antegrade cerebral perfusion

## Abstract

**Background:**

In this case we discuss the management of a pediculated floating thrombus in the aortic arch which led to peripheral embolization and acute ischemia oft he left leg.

**Case presentation:**

A healthy 46 year old female patient presented with pain in her left leg and progressive numbness. Computed Tomography Angiography (CTA) showed an acute ischemia of the left leg (Rutherford 2 B) with a 2 cm thrombus distal of the aortic bifurcation. Emergency operation with embolectomy, selective thrombembolectomy and patch plasty on the tibioperoneal trunk and local lysis was performed. As part of a further diagnostic examination a thoracic CT scan has been performed revealing a pediculated-floating 2 cm thrombus in the aortic arch. Four days after the initial operation thrombus excision via a minimally invasive access way has been performed. After initiation of the extracorporeal circulation, selective unilateral antegrade cerebral perfusion has been established in mild (30–32 °C) systemic hypothermia. Patients postoperative course was uneventful. Histological evaluation of the mass demonstrated thrombotic material without evidence of infection or malignacy.

**Conclusion:**

A pediculated spontaneous thrombus may develop in aortic arch in patients without traditional risk factors or family history of embolic events. Two stage operation was feasible and safe.

## Introduction

Peripheral embolization of a thrombus fragment located in the aortic arch is a rare finding in clinical practice. In non-aneurysmal vessels but especially in young patients with no history of atherosclerosis this condition becomes rare. In the current manuscript we aimed to report and discuss the surgical management of a pediculated-floating 2 cm thrombus in the aortic arch which already lead to an acute ischemia of the left leg (Rutherford 2 B). Procedural steps were initiated after interdisciplinary decision making in the Cardio-Vascular-Surgery Team.

## Case

**Fig. 1:**
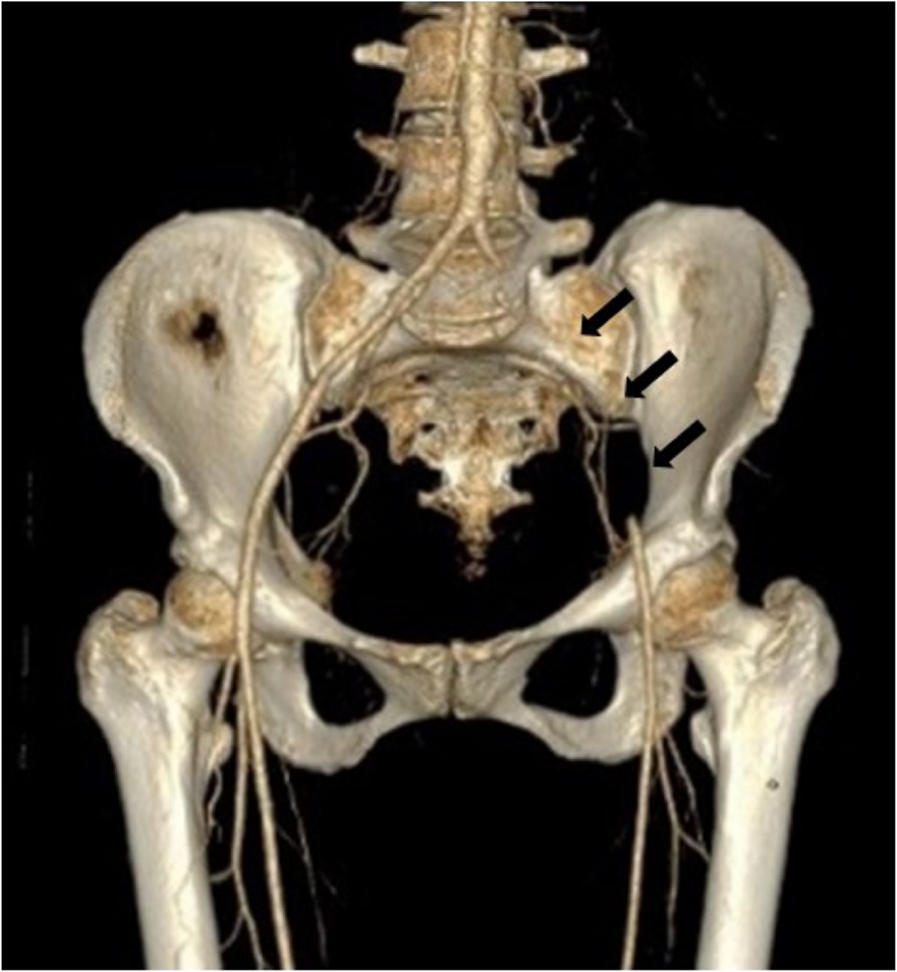
Computed Tomography Angiography showing occlusion of the left sided iliac artery distal of the aortic bifurcation (triple arrow)

**Fig. 2:**
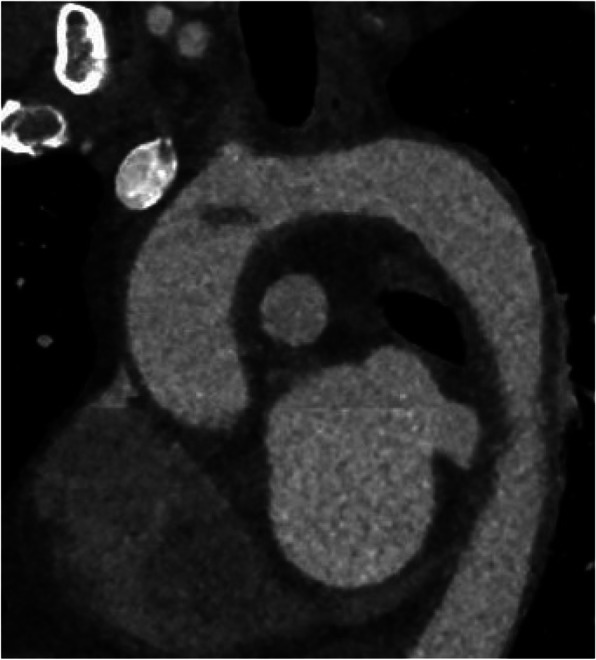
Thoracic computer tomography showing a pediculated-floating 2 cm thrombus (arrow) in the aortic arch (zone 0), representing the origin of the peripheral embolization

A 46 year old female patient presented with pain in her left leg and progressive numbness. She has no history of valvular heart disease, atrial fibrillation, hypercoagulable disorder, peripheral vascular disease or claudication. Due to absent pedal pulse an emergency Computed Tomography Angiography (CTA) was initiated with diagnosis of an acute ischemia of the left leg (Rutherford 2 B) with a 2 cm thrombus distal of the aortic bifurcation (Fig. 1). Emergency operation via left inguinal access and additional Szilagyi access with embolectomy, selective thrombembolectomy and patch plasty on the tibioperoneal trunk and local lysis was performed. As part of a further diagnostic examination, a thoracic CT scan has been performed revealing a pediculated-floating 2 cm thrombus in the aortic arch (zone 0, Fig. 2). The remaining aorta was free from atherosclerosis or aneurysmal disease. Postoperative transthoracic echocardiography was without pathological findings.

**Fig. 3:**
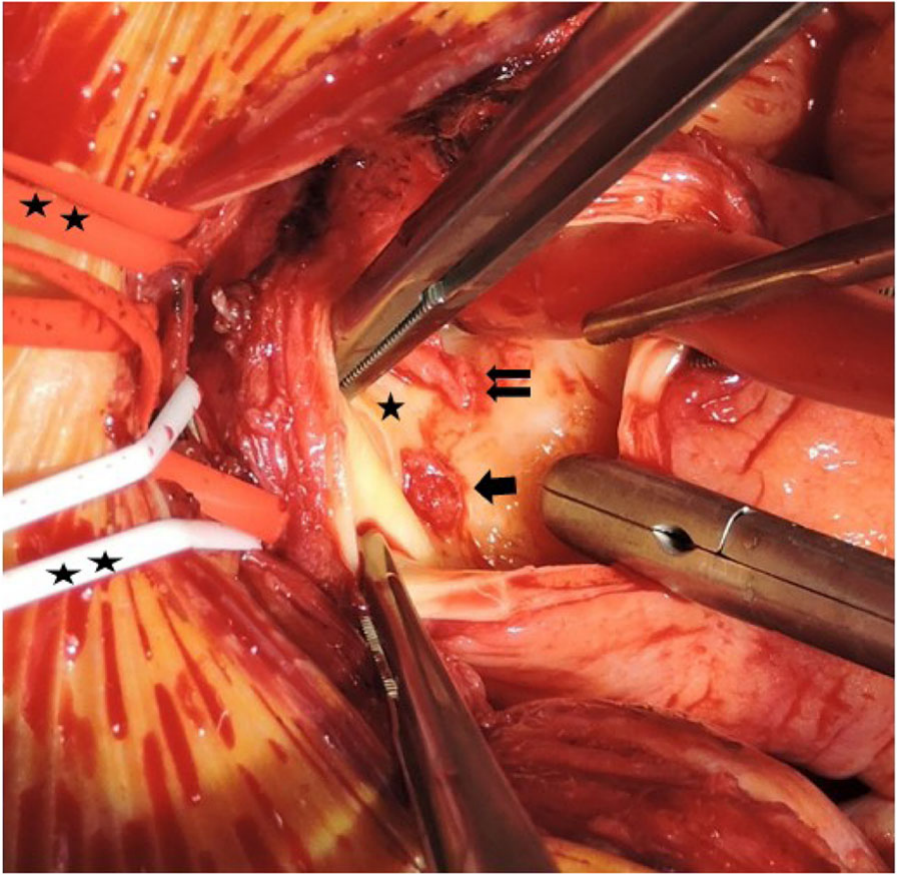
Surgical view of a longitudinal incision of the distal ascending aorta and direct visualization of a 2 cm thrombus (single arrow) and a smaller 3 mm thrombus (double arrow) located below the origin of brachicephalic trunk (single star) and the origin of the left common carotid artery respectively. Brachiocephalic trunk and left common carotid artery are looped and marked by double star. The last loop contains left brachiocephalic vein

Four days after the initial operation thrombus excision via a minimally invasive access way (partial upper sternotomy) was performed. Basic operative steps were as follows: Limited skin incision (8 cm) followed by left sided L-shaped partial upper sternotomy beginning from jugulum diverted to 4th intercostal space. Direct arterial cannulation via right axillary artery and venous cannulation through the right atrium. After initiation of the extracorporeal circulation, selective unilateral antegrade cerebral perfusion was established in mild (30–32 °C) systemic hypothermia under control of brain tissue oxygen saturation with near-infrared spectroscopy (INVOS). Longitudinal incision of the distal ascending aorta, direct visualization and excision of the 2 cm thrombus (Fig. 3) and a smaller 3 mm thrombus in the small curvature has been performed. The aortic wall showed no local dissection, hematoma or atherosclerotic changes. Direct closure of the aortotomy has been performed using 4/0 Prolene. Duration of isolated antegrade cerebral perfusion was 8 min, extracorporeal circulation time counted 36 min in total. Patients postoperative course was uneventful. Thus intensive care unit and hospital stay counted 36 h and 13 days respectively. After careful consideration within the multidisciplinary team of the operating surgeons and hematologist an antiplatelet therapy with aspirin and additional anticoagulation with phenprocoumon was established postoperatively.

Histological evaluation of the mass demonstrated thrombotic material without evidence of infection or malignancy.

After 20 days of cardiac rehabilitation, a tumor screening via positron emission tomography (PET) scan was negative. Long-term electrocardiogram (ECG) monitoring showed no atrial fibrillation or any other pathologic finding. The patient was referred for hematology consultation with negative results for congenital or acquired thrombophilia. Paraproteins were excluded, there were no Janus-Kinase-2 (JAK-2) mutations. Antithrombin and functional Protein C were normal. During therapy with phenprocoumon, Protein S was lower so that hereditary Protein S deficiency cannot be fully excluded. Oral anticoagulants are continued while Aspirin was stopped due to a resistance. In the clinical follow up the patient presented in a good clinical condition. CT scan 1 year postoperative showed no further embolic events or remaining embolic material in the aorta.

## Discussion

Spontaneous Aortic Arch thrombus is a rare but previously in literature described origin for peripheral embolization [1]. Common causes leading to aortic thrombus formation are congenital or acquired blood disorders, tumor, inflammation, hormone therapy, steroid use or structural abnormalities in the aortic wall, myxoma, fibroelastoma as well as persistent foramen ovale [2]. Once none of these causes were applicable in our case, we put the focus on Behçet- Adamantiades-Syndrom especially due to patient’s mediterranean heritage. This rare inflammatory disorder, which affects multiple organ systems, may also lead to aortic thrombus formation. This diagnosis could not be confirmed, thus etiology of aortic thrombus remains ambiguous in our case. This is not an uncommon scenario once in the largest series of 23 patients with mobile thrombi of the aortic arch published from Laperche et al. back in to 1997 the etiology of the aortic thrombus formation remains in 83% of patient cohort unclear [3].

Thrombus in the ascending aortic or aortic arch can be associated with embolic events with a subsequent cerebral, visceral and peripheral ischemia. The latter is decisive for the clinical appearance of the patient, which can vary. Once peripheral emboli is retrieved and the peripheral circulation is restored patients without an obvious etiology for the embolic event warrant urgent hypercoagulable screening and diagnostic imaging, including CT-Scan of the thoracic and abdominal aorta as well as echocardiography, to rule out pathology of the thoracic aorta as well as intracardiac thrombus.

Medical treatment including heparinization, endovascular stenting, or surgery have been proposed according to the International Guidelines for the treatment of mobile aortic thrombosis [4] but no comparative data are available. According to the published data location of thrombus strongly affects the possible therapeutic strategies. While thrombi in the abdominal aorta or its branches are preferable treated conservatively [5, 6] endovascular stenting is considered in cases involving the descending or abdominal aorta [7]. Once intraluminal thrombus in the ascending aorta or aortic arch represent a contraindication for the new evolving stent graft devices mortality and morbidity of a cardiosurgical approach and thrombus-removal have to be weight against the risk of distal or even vast cerebral embolization that would remain with conservative anticoagulation therapy.

Given the concern for potential cerebral and recurrent peripheral embolization, we decided against conservative management with anticoagulant agents alone and pursued urgent surgical exploration via a minimally invasive access way. Since the thrombus was pediculated catheterbased thrombectomy was not discussed in this case.

Our standard surgical approach for aortic arch surgery has been described previously in detail [8]. Main steps include direct axillary cannulation and moderate-to-mild systemic hypothermia during selective antegrade cerebral perfusion. Dissection of the aortic arch and control of supraaortic arch branches can be carried out via partial upper sternotomy, which may reduce surgical trauma. In our case, longitudinal aortotomy with primary thrombus removal and repair of aortic wall is preferred instead of interposition graft replacement, given the theoretical risk of prosthetic material.

Aligned with actuarial literature initiation of systemic anticoagulation with phenprocoumon or antiplatelet therapy with aspirin should be considered as secondary prophylaxis for recurrent emboli [9]. Aspirin that has been postoperatively initiated in our case was stopped due to a resistance detected in Multi Plate testing (Multiplate®Analyzer, Roche, Basel, Switzerland) leading to a suggestion of lifelong anticoagulation with phenprocoumon as secondary prophylaxis with an internationalized ratio between 2.5 and 3.

## Conclusion

A pediculated spontaneous thrombus may develop in aortic arch in patients without traditional risk factors or family history of embolic events.

Two-step surgical treatment of the peripheral acute ischemia followed by minimally invasive thrombectomy in the aortic arch performing through a partial upper sternotomy can be performed safely in a setting of mild systemic hypothermia during selective unilateral antegrade cerebral perfusion.

## Data Availability

Not applicable.

## Abbreviations

CTA: Computed Tomography Angiography
CT: Computed Tomography
INVOS: Near-infrared spectroscopy
PET: Positron emission tomography
ECG: Electrocardiogram
JAK-2: Janus-Kinase 2
